# Radiomics and quantitative multi-parametric MRI for predicting uterine fibroid growth

**DOI:** 10.1117/1.JMI.11.5.054501

**Published:** 2024-09-12

**Authors:** Karen Drukker, Milica Medved, Carla B. Harmath, Maryellen L. Giger, Obianuju S. Madueke-Laveaux

**Affiliations:** a University of Chicago, Department of Radiology, Chicago, Illinois, United States; b University of Chicago, Department of Obstetrics and Gynecology, Chicago, Illinois, United States

**Keywords:** uterine fibroids, women’s health, machine learning, artificial intelligence, radiomics, multi-parametric magnetic resonance imaging

## Abstract

**Significance:**

Uterine fibroids (UFs) can pose a serious health risk to women. UFs are benign tumors that vary in clinical presentation from asymptomatic to causing debilitating symptoms. UF management is limited by our inability to predict UF growth rate and future morbidity.

**Aim:**

We aim to develop a predictive model to identify UFs with increased growth rates and possible resultant morbidity.

**Approach:**

We retrospectively analyzed 44 expertly outlined UFs from 20 patients who underwent two multi-parametric MR imaging exams as part of a prospective study over an average of 16 months. We identified 44 initial features by extracting quantitative magnetic resonance imaging (MRI) features plus morphological and textural radiomics features from DCE, T2, and apparent diffusion coefficient sequences. Principal component analysis reduced dimensionality, with the smallest number of components explaining over 97.5% of the variance selected. Employing a leave-one-fibroid-out scheme, a linear discriminant analysis classifier utilized these components to output a growth risk score.

**Results:**

The classifier incorporated the first three principal components and achieved an area under the receiver operating characteristic curve of 0.80 (95% confidence interval [0.69; 0.91]), effectively distinguishing UFs growing faster than the median growth rate of $0.93\;{\mathrm{cm}}^{3}/\mathrm{year}/\mathrm{fibroid}$ from slower-growing ones within the cohort. Time-to-event analysis, dividing the cohort based on the median growth risk score, yielded a hazard ratio of 0.33 [0.15; 0.76], demonstrating potential clinical utility.

**Conclusion:**

We developed a promising predictive model utilizing quantitative MRI features and principal component analysis to identify UFs with increased growth rates. Furthermore, the model’s discrimination ability supports its potential clinical utility in developing tailored patient and fibroid-specific management once validated on a larger cohort.

## 1 Introduction

Uterine fibroids (UFs) represent a prevalent health concern among women of reproductive age, with often a substantial impact on their quality of life. UFs are benign soft tissue tumors of the uterus that affect up to 80% of women in the United States. These benign tumors, originating from the smooth muscle cells of the uterine wall, can manifest with a spectrum of symptoms, including pelvic pain, abnormal bleeding, and reproductive dysfunction, with symptoms ranging from mild to severe.^1^ The management of UFs is multifaceted, ranging from conservative approaches such as medication or lifestyle modifications to surgical interventions, each tailored to the individual patient’s needs.^2^

The integration of radiomics and multi-parametric magnetic resonance imaging (MRI) may aid in the understanding and management of UFs. Radiomics, the extraction and analysis of many quantitative features from medical images, empowers researchers with a comprehensive characterization of tissue properties beyond what is discernible through traditional human imaging assessment. In conjunction with multi-parametric MRI, which incorporates various imaging sequences to provide a holistic view of tissue characteristics, these technologies hold promise in revealing nuanced characteristics of UFs.

This study aimed to explore the utilization of radiomics and quantitative multi-parametric MRI for the prediction of UF growth and was a complete do-over of an earlier version presented at the 2024 SPIE Medical Imaging conference.^3^ We sought to identify robust imaging biomarkers that could serve as predictors for the trajectories of UF development. Such predictive capabilities could potentially enhance clinical decision-making, allowing for timely interventions and personalized treatment strategies.

## 2 Material and Methods

### 2.1 Patient Cohort and Image Acquisition

In this study, we retrospectively analyzed data from an earlier prospective pilot study, conducted from September 2019 to October 2022 and approved by the University of Chicago Medicine Institutional Review Board (IRB18-1361). Female patients from the University of Chicago women’s clinic were recruited based on eligibility confirmed through chart review. The study group comprised premenopausal English-speaking patients, aged over 18 years, with ultrasound-confirmed diagnosis of at least one UF larger than 2 cm. Exclusion criteria included immediate intervention requirement, pregnancy, breastfeeding, receiving gonadotropin-releasing hormone, or inability to undergo contrast-enhanced MRI. Two study visits, at least 1 year apart (mean: 16 months, range: 11.5 to 29.5 months), were conducted. Patient medical, surgical, and gynecological history and demographics and health/lifestyle characteristics were collected.^4^

A pelvic MRI was obtained at each visit. MRI was conducted using a 3T dStream Philips Ingenia scanner. The comprehensive three-part imaging protocol included (1) standard clinical pelvic MRI non-contrast sequences; (2) non-contrast-enhanced quantitative sequences focused on the uterus (mp-qMRI), including a 2D SAG T2 map, 2D SAG T2*/R2* map, and SAG diffusion weighted imaging, providing the apparent diffusion coefficient (ADC) map; and (3) a contrast agent-enhanced sequence including SAG 3D T1 mDIXON uterus-only dynamic contrast-enhanced MRI (DCE-MRI) sequence.

**Fig. 1:**
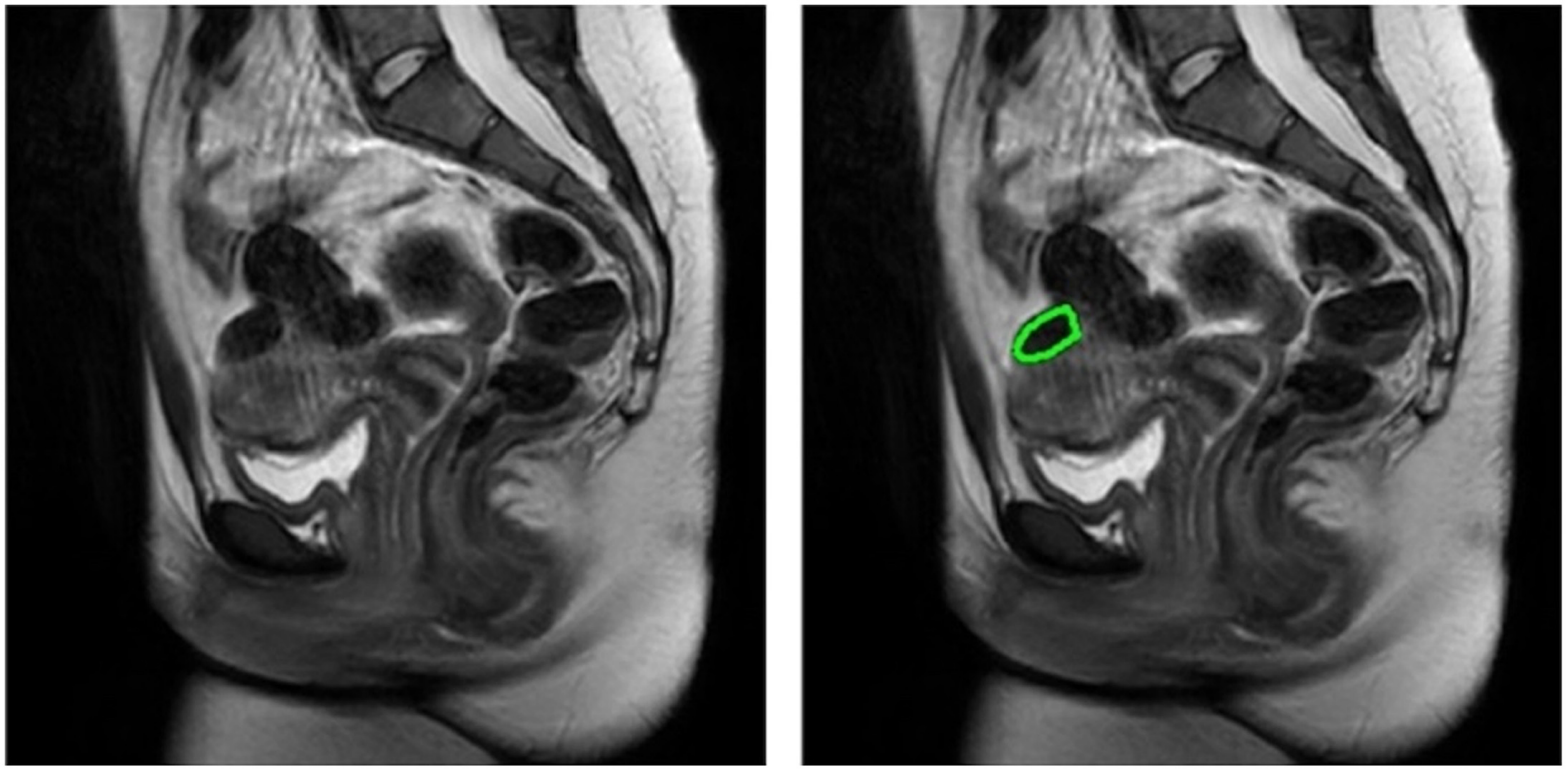
Example T2 image of a uterine fibroid without and with the expert contour (in green).

An experienced radiologist manually outlined up to three UFs on all the standard clinical T2-weighted sequences for subsequent tracing on the mp-qMRI sequences (Fig. 1).

### 2.2 Methods

In this study, the task was to predict whether a UF would grow faster than the median growth rate based on the baseline exam only, with the growth rate for each UF assumed to be constant.

#### 2.2.1 Qualitative expert assessment features

Radiologists assessed qualitative morphological features: the presence of bright rim and signal intensity on T2, the presence of bright rim and signal intensity on ADC, and overall, rim, and core enhancement features on DCE-MRI. A total of 10 qualitative features were investigated, with some being binary (e.g., yes/no rim enhancement) and some being categorical (Table S1 in the Supplementary Material).

#### 2.2.2 Quantitative features

UF volumes were estimated from the area of the UF at the largest cross-section, as traced on the SAG T2 images approximating the UF cross-sections as ellipses and UFs as ellipsoids. For four patients who did not complete the second visit, the volume was estimated on pre-surgery MRI or ultrasound, respectively.^4^ The UF growth rate was determined as the difference in UF volume (cubic centimeter) divided by the elapsed time (years) between the two exams. The growth rate was assumed to be constant.

All features were calculated from the baseline exam. The mp-qMRI average T2, T2*, R2*, and ADC values over the UF regions of interest served as the first four features. In addition, 40 morphological and textural radiomics features were calculated for the UFs outlined on the most representative 2D image slice of the T2 and ADC mapping. These features included 12 morphological features such as volume, circularity, eccentricity, minor- and major axis length, solidity, perimeter, and 28 gray-level co-occurrence matrix (GLCM) texture^5^^–^^7^ features (14 each derived from the T2 and ADC maps) of the UFs. The total number of initial quantitative features was 44 (Table S2 in the Supplementary Material).

#### 2.2.3 Radiomics growth risk score

The task of interest was to predict whether a UF was expected to exhibit a “faster” growth rate (i.e., faster than the median growth rate in the cohort). We investigated the performance of individual quantitative features in this task. Rather than performing supervised feature selection within the leave-one-fibroid-out analysis, which would have likely been very unstable due to the small dataset and a large number of features relative to the dataset size, we used an unsupervised technique to reduce dimensionality before input to the classifier. For this purpose, principal component analysis was used to reduce the dimensionality of the initial 44 quantitative features to the smallest number of principal components that captured 97.5% of the variance within the dataset. These principal components were then used as input to a linear discriminant analysis classifier. This classifier was trained and tested within a leave-one-fibroid-out fashion to predict which UFs would grow faster than the median growth rate within the cohort.

#### 2.3.4 Statistical analysis

The performance of the qualitative expert assessment features was evaluated using the area under the receiver operating characteristic (ROC) curve (${\mathrm{AUC}}_{\text{ROC}}$) sensitivity, specificity, and positive predictive value. For the performance analysis of the quantitative features and the radiomics growth risk score, ${\mathrm{AUC}}_{\text{ROC}}$ served as the main performance metric in the task of distinguishing UFs growing faster or slower than the median growth rate, respectively. The area under the precision-recall curve, ${\mathrm{AUC}}_{\mathrm{PR}}$, and the hazard ratio from time-to-event analysis (using the linearly interpolated or extrapolated time to grow $1\;{\mathrm{cm}}^{3}$ assuming a constant growth rate) served as adjunct metrics.

## 3 Results

### 3.1 Patient Cohort and Uterine Fibroids

**Fig. 2:**
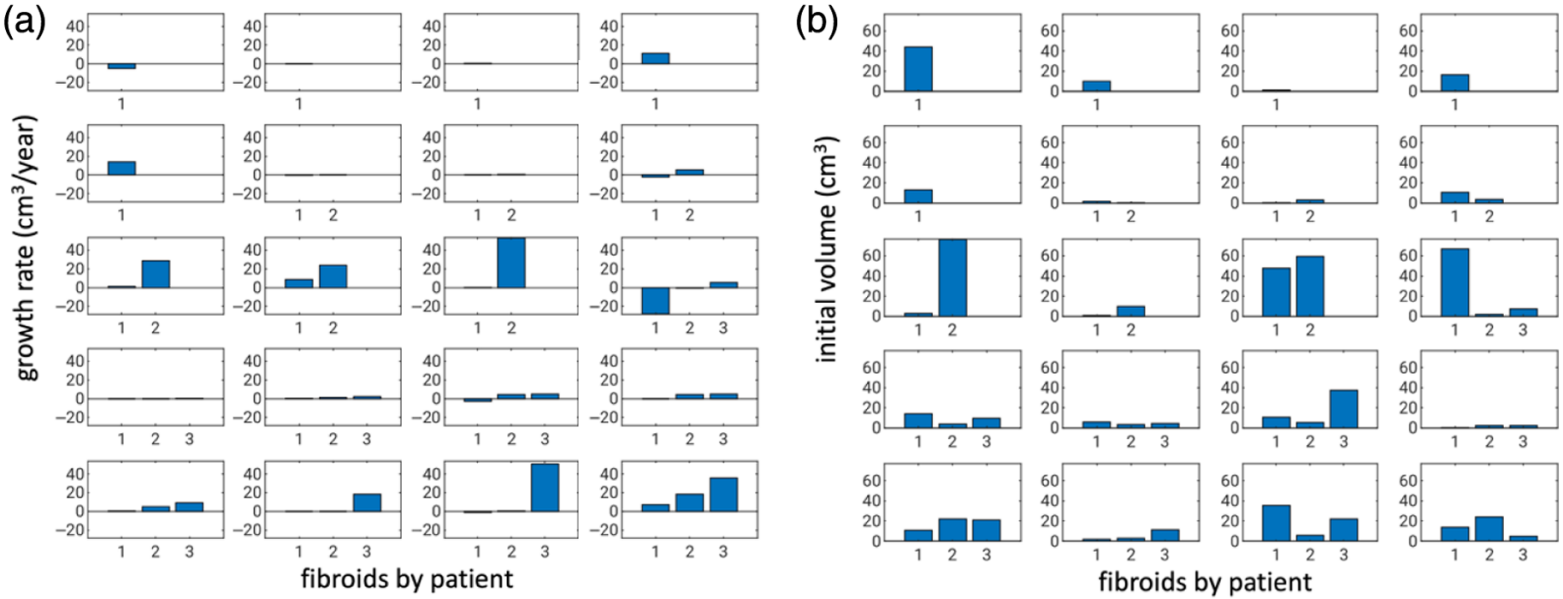
(a) Growth rates of each UF (N=44) for each patient ($N_{p\;}=20$) and (b) the initial estimated volume per UF. The patients were first ordered by increasing the number of fibroids and then by total fibroid burden growth, and fibroids for a given patient were ordered by the increasing growth rate. The ordering in panels (a) and (b) is the same. Note that a negative growth rate indicates regression (N=13).

A total of 66 UFs in 30 subjects (median age: 42 years) were identified through baseline quantitative MRIs. Follow-up information was available for a subset of 20 subjects (44 UFs), and this subset was included in our study (Fig. 2). The average follow-up time was 487 days, median 404 days, and range 345 to 894 days. The median UF growth rate was $0.93\;{\mathrm{cm}}^{3}/\mathrm{year}/\mathrm{fibroid}$, a 29% relative increase in volume. Of the UFs, 30% (13/44) decreased in size over time, whereas 20% (9/44) demonstrated a rapid growth rate of more than $10\;{\mathrm{cm}}^{3}$ per year. The UFs that diminished in size had a median regression (shrinkage) rate of $-0.40\;{\mathrm{cm}}^{3}/\mathrm{year}/\mathrm{fibroid}$ (a 13% relative decrease in volume). For those UFs that increased in size, the median growth rate was $5.05\;{\mathrm{cm}}^{3}/\mathrm{year}/\mathrm{fibroid}$ (a 53% relative increase in volume). Note that UFs within one patient did not necessarily exhibit the same growth behavior, i.e., the growth rates did not appear to be highly correlated, and the growth behavior did not appear to depend strongly on the initial size (Fig. 2).

### 3.2 Growth Risk Prediction

#### 3.2.1 Qualitative expert assessment features

**Fig. 3:**
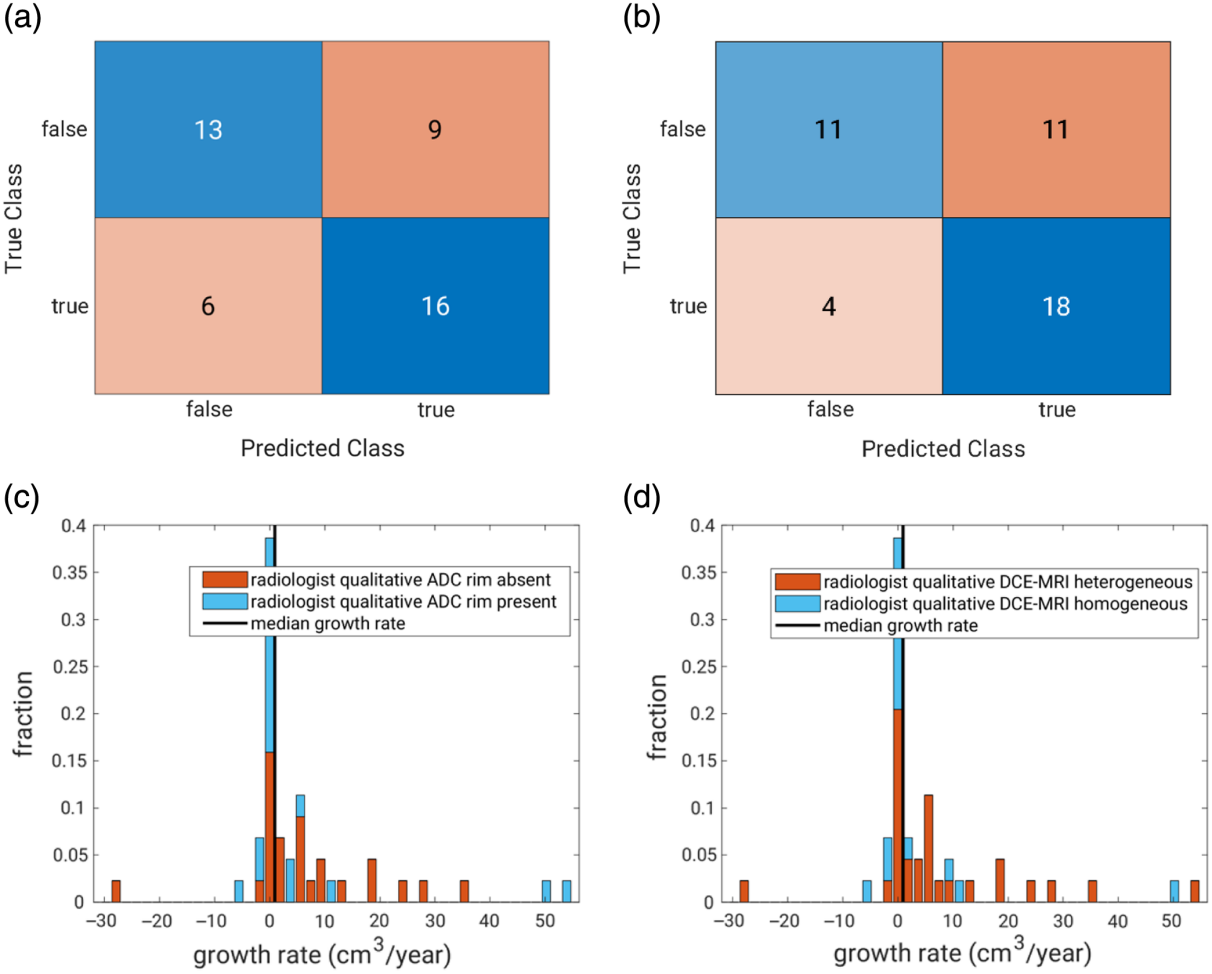
Confusion matrices for (a) the qualitative features “rim presence on ADC” and (b) “heterogeneous enhancement on DCE-MRI” in predicting fibroid growth as faster or slower than the median (“true” = “faster” and “false” = “slower” growth). Stacked histograms of the actual growth rates color coded using the radiologist qualitative features of (c) “rim presence on ADC” and (d) “heterogeneous enhancement on DCE-MRI.” In an “ideal” situation, the bars to the left of the vertical line indicating the median growth rate would be blue, and all to the right would be orange.

Two of the 10 examined qualitative features showed some promise in identifying UFs that grew faster than the median growth rate (Table 1 and Fig. 3). Note that these features were binary (yes/no), so their ROC curves only had a single operating point [other than (0,0) and (1,1)], making the AUC a rough estimate. Slower-growing UFs were more likely to present with an enhancing rim on the ADC map, and faster-growing UFs were more likely to display heterogeneous contrast-agent uptake on DCE-MRI (Fig. 3).

**Table 1:** Performance of the expert-radiologist assessed features in the task of predicting which fibroids would grow faster than the median growth rate. The square brackets indicate 95% CIs.

	AUC	Sensitivity	Specificity	Positive predictive value
Rim presence on ADC	0.66 [0.51; 0.79]	73% [55%; 91%]	59% [36%; 77%]	64% [52%; 78%]
Heterogeneous uptake on DCE-MRI	0.66 [0.53; 0.79]	82% [64%, 95%]	50% [27%; 73%]	62% [52%; 74%]

#### 3.2.2 Quantitative features

**Fig. 4:**
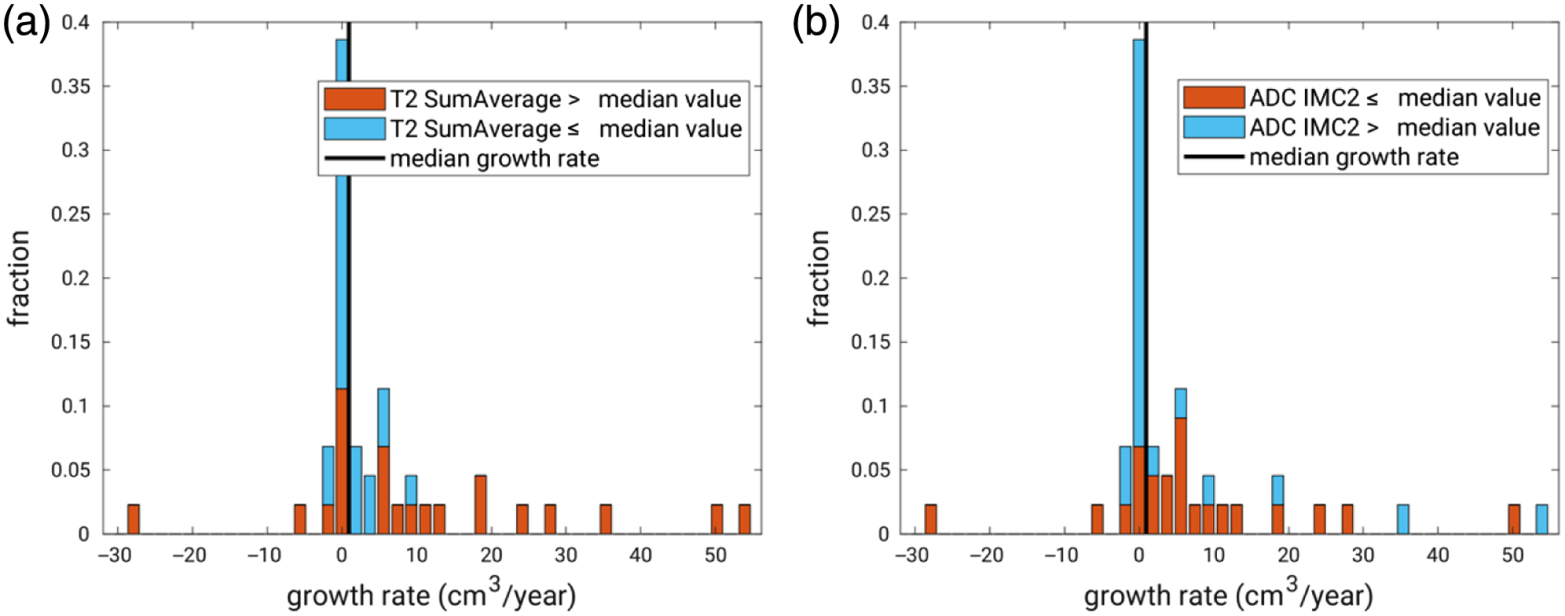
Stacked histograms of the actual growth rates color coded using (a) the GLCM texture feature “sum average” derived from the T2 map and (b) the GLCM texture feature IMC2 derived from the ADC map. In an “ideal” situation, the bars to the left of the vertical line indicating the median growth rate would be blue, and all to the right would be orange.

None of the morphological features on their own exhibited the potential to distinguish between faster- and slower-growing UFs. Two GLCM texture features derived from the T2 map exhibited modest predictive ability: the informational measure of correlation feature 1 (IMC1, quantifying the complexity of texture using mutual information derived from the GLCM) and the sum average feature, SumAverage, measuring the relationship between occurrences of pairs with lower intensity values and occurrences of pairs with higher intensity values, with AUC values of 0.69 [0.51; 0.82], and 0.72 [0.57; 0.87], respectively. In addition, two of the GLCM texture features derived from the ADC map exhibited some predictive ability: IMC1 and the informational measure of correlation feature 2 (IMC2), with AUC values [95% confidence interval (CI)] of 0.69 [0.53; 0.84] and 0.70 [0.53; 0.86], respectively (Table 2). Fibroids with a larger than median SumAverage feature on the T2 map or a lower than median IMC2 feature on the ADC map were more likely to exhibit a faster growth rate (Fig. 4).

**Table 2:** Performance of the quantitative radiomics features and the radiomics growth risk score in the task of predicting which fibroids would grow faster than the median growth rate. The square brackets indicate 95% CIs. ${\mathrm{AUC}}_{\mathrm{ROC}}$, area under the ROC curve; ${\mathrm{AUC}}_{\mathrm{PR}}$, area under the precision-recall curve.

	${\mathrm{AUC}}_{\mathrm{ROC}}$	${\mathrm{AUC}}_{\mathrm{PR}}$
T2 IMC1	0.69 [0.51; 0.82]	0.69 [0.54; 0.85]
T2 SumAverage	0.72 [0.57; 0.87]	0.71 [0.53; 0.87]
ADC IMC1	0.69 [0.53; 0.84]	0.62 [0.43; 0.84]
ADC IMC2	0.70 [0.53; 0.86]	0.61 [0.40; 0.81]
Radiomics risk score	0.80 [0.69; 0.91]	0.79 [0.67; 0.90]

#### 3.2.3 Radiomics growth risk score

The first three principal components captured 97.5% of the variance and were used as input to the linear discriminant analysis classifier. In the principal component decomposition, the morphology features of perimeter length and UF extent received the highest weights as did the texture feature of variance on both the T2 and ADC maps. The linear discriminant analysis using the first three principal components as input in the leave-one-fibroid-out analysis achieved an AUC of 0.80 (95% CI: [0.69; 0.91]) in the task of predicting whether UFs would grow at a rate faster than the median within the cohort (Table 2). The corresponding area under the precision-recall curve was 0.79 [0.67; 0.90] (compared with the baseline random guessing value of 0.5 given that 50% of the UFs grew faster than the median).

**Fig. 5:**
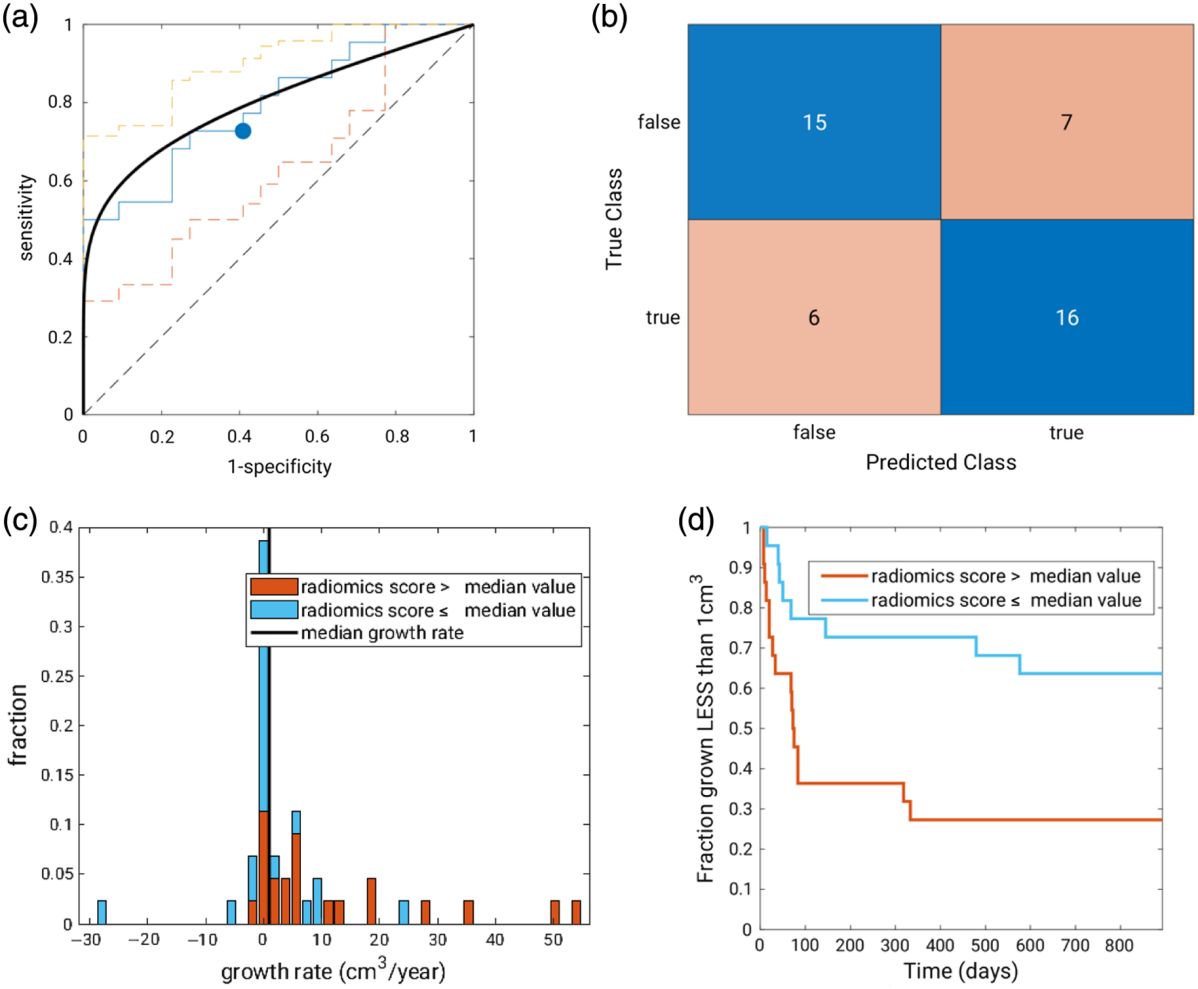
(a) ROC curve (“raw” in solid blue line, fitted using the proper binormal model^8^ in bold black line) and 95% CI (in dashed lines) with “raw” operating point (blue circle) corresponding to the median as threshold for the radiomics risk score decision variable, (b) confusion matrix corresponding to this operating point, (c) stacked histogram of the actual growth rates color coded using the radiomics risk score, and (d) time-to-event analysis based on dividing the cohort by median radiomics risk score (N=22 at time = 0 for each sub-cohort). Note that the radiomics model’s task was to predict growth “slower” or “faster” than the median not to estimate the actual growth rate.

In time-to-event analysis, dividing the cohort (N=44) based on the median growth risk score and assuming linear growth (constant growth rate), the median time to grow $1\;{\mathrm{cm}}^{3}$ for “higher risk” UFs, i.e., those with a radiomics risk score higher than the median score, was 74 days. Only 6 (27%) of the “higher risk” UFs would not have grown $1\;{\mathrm{cm}}^{3}$ by the maximum follow-up time (894 days). Most of the UFs receiving a radiomics risk score lower than the median score had not grown $1\;{\mathrm{cm}}^{3}$ by the maximum follow-up time. In fact, only 8 (36%) out of the 22 UFs in the “lower risk” group had grown more than $1\;{\mathrm{cm}}^{3}$ by that time. The quantitative radiomics risk score demonstrated potential clinical utility with the UFs receiving a lower than median radiomics risk score having a threefold reduction in odds of undergoing a larger than $1\;{\mathrm{cm}}^{3}$ actual growth within the study period (hazard ratio of 0.33 (95% CI: [0.15; 0.76]); Fig. 5).

## 4 Discussion and Conclusion

This pilot study successfully developed a predictive model utilizing quantitative MRI features and principal component analysis and highlights the potential of multiparametric quantitative MRI as a tool to predict UF growth. It is interesting to note that most qualitative radiologist-assessed features demonstrated limited predictive ability in our cohort. The presence of a “rim” has been found a useful sign in other studies. One study found that the presence of a “rim” on the T2 map was predictive of the response to high-intensity ultrasound ablation.^9^ In our exploration of qualitative radiologist-assessed features, we found that the presence of a “rim” on the ADC map was somewhat predictive of UF growth. In our quantitative explorations, we found that by incorporating quantitative MRI features and employing principal component analysis, the developed predictive model exhibited promising discrimination ability. The area under the ROC curve of 0.80 indicates that the model effectively distinguishes UFs growing faster than the median growth rate. Moreover, the hazard ratio of 0.33 (95% CI: [0.15; 0.76]) obtained from the time-to-event analysis suggests potential clinical utility in identifying UFs with decreased and increased growth risks.

UFs, known for their heterogeneous characteristics, pose challenges in accurate detection, diagnosis, and treatment prediction.^10^^–^^14^ Deep learning algorithms offer a promising avenue for enhancing diagnostic precision and efficiency.^15^ Addressing the complexities of preoperative differentiation between UFs and leiomyosarcomas is crucial, with innovative techniques such as AI algorithms showing promise.^16^ Although current efforts in radiomics and machine learning have predominantly focused on predicting treatment responses, particularly in the context of high-intensity focused ultrasound ablation, there remains a substantial need for broader applications.^10^^,^^17^^–^^19^

Cheng et al.^10^ and Zhou et al.^14^ have demonstrated the potential of radiomics models, primarily utilizing T2-weighted imaging (T2WI), in predicting ablation efficacy and facilitating preoperative screening. In addition, Li et al.’s^11^ work in interpretable MRI-based radiomics has contributed to a deeper understanding of fibroid heterogeneity. The importance of predicting postoperative reintervention risk for optimizing outcomes is highlighted by Qin et al.’s^13^ effective integration of clinical-imaging features and T2WI radiomics.

In our pilot study presented here, we evaluated the risk of rapid fibroid growth, essential for early intervention, using a radiomics model. Our growth rate observations align with the existing literature, showcasing varied rates of fibroid growth or regression over time.^20^ Prior studies have reported median growth rates ranging from 18% to 82% per year,^21^ and our relative growth rates fall within that window. Furthermore, studies on fibroid vascularization, growth dynamics, and racial disparities contribute valuable insights into their natural history and growth patterns.^22^^–^^24^ In a large study of patients with a single UF, Li et al.^20^ developed a linear regression model predicting fibroid growth based on clinical variables with promising results. Notably, Chen and Ward’s^24^ mathematical model, incorporating variables such as vascularity and estrogen levels, adds depth to our understanding of fibroid growth dynamics.

Our contribution lies in exploring specialized quantitative MR sequences and fusion methods for radiomics-based prediction of fast fibroid growth risk, which could complement existing clinical and radiomics models. These advancements underscore the potential of radiomics in enhancing prognostic assessment and treatment selection for UFs.^10^^–^^19^ However, further validation in larger cohorts and exploration of additional imaging modalities and biomarkers are warranted to comprehensively evaluate fibroid growth and treatment response, aligning with guidelines advocating for individualized treatment approaches.^25^ To confirm the efficacy of multiparametric quantitative MRI in identifying fast-growing UFs, future studies should consider obtaining T2, ADC, and UF volume data in a larger population. This would further validate the predictive model and provide valuable insights for better-informed disease management and improved clinical outcomes. By tailoring patient-specific management plans based on the growth potential of UFs, long-term cost savings could be achieved by reducing the rate of invasive interventions for slow-growing UFs and allowing for timely or preventative interventions in cases of fast-growing UFs. Such timely intervention results in increased access to minimally invasive surgical techniques and decreased hospitalizations for severe UF symptoms, acute anemia transfusions, and obstetric complication rates.

This study had several limitations with the first being the modest size of the cohort. This was because data were collected prospectively, and the study required patients to undergo additional research multi-parametric quantitative MRI scans ∼16 months apart, not to mention that the study period was interrupted by the COVID-19 global pandemic. Another limitation stemmed from the modest cohort size, prompting us to address the prediction of UF growth as a classification problem rather than a regression one, which would have been more powerful. Last, due to the small size of the cohort and outliers such as nine rapidly growing fibroids and one substantially shrinking fibroid, developing a robust cross-validated regression model predicting actual growth rates proved infeasible.

We obtained promising results in the prediction of which UFs in the cohort would demonstrate an increased growth rate. However, further validation on a larger cohort is necessary to establish the reliability and effectiveness of the predictive model in clinical practice. Achieving this could help lead to the development of tailored management strategies for UFs and improved patient outcomes.

## Supplementary Material



## Data Availability

The data used in this paper are not yet publicly available.
