# Supplementary material for: Radiomics and quantitative multi-parametric MRI for predicting uterine fibroid growth
Source: J Med Imaging (Bellingham). 2024 Sep 12;11(5):054501. doi: 10.1117/1.JMI.11.5.054501 (PMC11391479; doi:10.1117/1.JMI.11.5.054501)
Supplement: Supplementary file 1 [file JMI_011_054501_SD001.docx]

**Table S1** Description of qualitative radiologist-assessed features

| **Feature** | **Description** | **Values** |
| --- | --- | --- |
| **T2** |  |  |
| **presence of bright rim** | Hyperintense rim surrounds the fibroid, over greater than 180° in any one image on T2-weighted MRI in the sagittal or axial plane | 0 (no),  1 (yes) |
| **signal intensity** | T2-weighted signal relative to the uterine parenchyma | -1 (hypo-),  0 (iso-),  1 (hyper-intense) |
| **ADC** |  |  |
| **presence of bright rim** | Hyperintense rim surrounds the fibroid, over greater than 180° in any one image on the ADC map in the sagittal or axial plane | 0 (no),  1 (yes) |
| **signal intensity** | ADC map values relative to the uterine parenchyma | -1 (hypo-),  0 (iso-),  1 (hyper-intense) |
| **DCE-MRI** |  |  |
| **Well defined enhancement** | Well-visualized fibroid in relationship to the adjacent uterine parenchyma | 0 (no),  1 (yes) |
| **Heterogeneous enhancement** | Greater than 50% of the fibroid demonstrates inhomogeneous enhancement following contrast-agent on early and equilibrium phases | 0 (no),  1 (yes) |
| **Enhancing rim** | Rim of hyperenhancement in relationship to the fibroid and adjacent myometrium, greater than 180° in any single image on the sagittal plane on the DCE-MRI sequences | 0 (no),  1 (yes) |
| **Dark rim** | Rim of hypo-enhancement in relationship to the fibroid and adjacent myometrium, greater than 180° in any single image on the sagittal plane on the DCE-MRI sequences | 0 (no),  1 (yes) |
| **Core enhancement signal** | Signal intensity in the fibroid core relative to the myometrium on early and equilibrium phases | -1 (hypo-),  0 (iso-),  1 (hyper-intense) |
| **Core enhancement timing** | Timing of fibroid core enhancement relative to the adjacent myometrium | -1 (late),  0 (same),  1 (early) |

ADC – apparent diffusion coefficient; DCE-MRI – dynamic contrast-enhanced MRI

**Table S2** Description of the quantitative radiomics features

| **Feature** | | **Description** |
| --- | --- | --- |
| **Quantitative MR sequence** | |  |
|  | **T2** | Derived from the T2 map for the fibroid region |
|  | **ADC** | Derived from the ADC map for the fibroid region |
|  | **T2^*^** | Derived from the T2^*^ map for the fibroid region |
|  | **R2^*^** | Derived from the R2^*^ map for the fibroid region |
| **Morphology** | |  |
|  | **Area** | Area of fibroid within most representative image slice (mm^2^) |
|  | **Volume** | Fibroid volume estimated from its area and ellipsoidal approximation in the z-direction (mm^3^) |
|  | **Major axis length** | Length of the major axis of the ellipse that has the same normalized second central moments as the region (mm) |
|  | **Minor axis length** | Length of the minor axis of the ellipse that has the same normalized second central moments as the region (mm) |
|  | **Circularity** | Roundness calculated as (4*Area*pi)/(Perimeter^2^) |
|  | **Eccentricity** | Eccentricity of the ellipse that has the same second-moments as the region, calculated as the ratio of the distance between the foci of the ellipse and its major axis length |
|  | **Orientation** | Orientation of the ellipse with the same second-moments as the region |
|  | **Euler number** | Number of objects in the region minus the number of holes in those objects |
|  | **Equivalent diameter** | Diameter of a circle with the same area as the region, computed as sqrt(4*Area/pi) |
|  | **Solidity** | Proportion of the pixels in the convex hull that are also in the region |
|  | **Extent** | Ratio of the number of pixels in the region to the number of pixels in the bounding box |
|  | **Perimeter** | Length of perimeter (mm) |
| **Texture from GLCM, derived for the T2 and ADC maps** | | |
|  | **Contrast** | The local variations in the gray-level co-occurrence matrix; measure of the intensity contrast between a pixel and its neighbors |
|  | **Correlation** | The joint probability occurrence of the specified pixel pairs; how correlated a pixel is to its neighbors |
|  | **Difference entropy** | Randomness/variability in neighborhood intensity value differences |
|  | **Difference variance** | Heterogeneity that places higher weights on differing intensity level pairs that deviate more from the mean |
|  | **Energy** | The sum of squared elements in the GLCM, also known as uniformity or the angular second moment |
|  | **Entropy** | The randomness/variability in the neighborhood intensities |
|  | **Homogeneity** | The closeness of the distribution of elements in the GLCM to the GLCM diagonal |
|  | **IMC1** | Informational measure of correlation 1; the correlation between the probability distributions quantifying the complexity of the texture) using mutual information |
|  | **IMC2** | Informational measure of correlation 2; the correlation between the probability distributions quantifying the complexity of the texture) using mutual information |
|  | **MaxCC** | Complexity of the texture |
|  | **SumAverage** | Relationship between occurrences of pairs with lower intensity values and occurrences of pairs with higher intensity values |
|  | **SumEntropy** | Sum of neighborhood intensity value differences |
|  | **SumVariance** | Measure of groupings of voxels with similar gray-level values |
|  | **Variance** | The mean of the squared distances of each intensity value from the mean value |

ADC – apparent diffusion coefficient, GLCM – gray-level co-occurrence matrix
